# Reducing MSSA Invasive Infection and Transmission in the NNICU using Whole Genome Sequencing and Environmental Surveillance.

**DOI:** 10.1017/ash.2025.232

**Published:** 2025-09-24

**Authors:** Tom Murray, Jayson Wright, David Peaper, Matthew Bizzarro, Noa Fleiss, Kathy Krechevsky, Jose Rivera-Vinas, Reese Hitchings, Thomas Durant

**Affiliations:** 1 Yale New Haven Children’s Hospital; 2 Yale School of Medicine; 3 Yale University School of Medicine; 4 Yale University; 5 Yale New Haven Hospital

## Abstract

**Background:** A Quality Improvement (QI) initiative to reduce invasive Staphylococcus aureus (SA) infections in a level IV neonatal intensive care unit (NICU) successfully eliminated Methicillin-resistant (MRSA) but not Methicillin-susceptible (MSSA) infections. A combination of SA whole genome sequencing (WGS) and environmental culturing helped to better understand the epidemiology of MSSA colonization and infection in the NICU and drive new infection prevention interventions. **Methods:** Environmental surveillance of high-touchpoint surfaces for SA was performed using Dey and Engley neutralizing agar. Selected isolates were confirmed as SA using Columbia Sheep’s Blood agar and Staphaurex testing. Statistical analyses examined correlations between monthly effective cleaning, hand hygiene compliance, and colonization rates. To better understand MSSA spread in the NICU, WGS was performed on a convenience sample of 42 MSSA isolates, sampled one month before and after an invasive MSSA infection. Data extracted from electronic health records were used for retrospective room tracing of colonized patients with related isolates to determine modes of transmission. **Results:** WGS analysis MSSA isolates revealed four MSSA strains from 29 patients suggesting within unit transmission, while 13 patients were colonized with unique MSSA isolates suggesting external sources. Retrospective room tracing of colonized patients identified three transmission patterns: subsequent room occupant transmission, intra-pod spread, and inter-pod transmission without patient transfer, with evidence that these strains were endemic within the unit for at least 3-12 months. Statistical analyses showed no significant correlation between environmental cleaning or hand hygiene compliance and colonization rates. **Conclusions:** Persistent MSSA colonization and invasive infections in the NICU result from both within-unit transmission and the introduction of unique isolates. These findings are being used to inform the development of new interventions, including updated below-the-elbow hand hygiene protocols, revised environmental cleaning plans, nurse-parent communication training, and a virtual reality hand hygiene training program for parents and staff. WGS of pathogenic organisms is a useful tool to drive QI initiatives aimed at reducing hospital-acquired infections.

